# Overexpression of PHRF1 attenuates the proliferation and tumorigenicity of non-small cell lung cancer cells

**DOI:** 10.18632/oncotarget.11842

**Published:** 2016-09-02

**Authors:** Yadong Wang, Haiyu Wang, Teng Pan, Li Li, Jiangmin Li, Haiyan Yang

**Affiliations:** ^1^ Department of Toxicology, Henan Center for Disease Control and Prevention, Zhengzhou 450016, China; ^2^ Henan Collaborative Innovation Center of Molecular Diagnosis and Laboratory Medicine, Xinxiang Medical University, Xinxiang 453003, China; ^3^ Department of Epidemiology, School of Public Health, Zhengzhou University, Zhengzhou 450001, China

**Keywords:** PHRF1, lung cancer, H1299 cell, tumorigenicity, cell cycle

## Abstract

The aim of this study was to investigate the potential role of PHRF1 in lung tumorigenesis. Western blot analysis was used to detect the expression of proteins. Quantitative reverse transcriptase polymerase chain reaction, immunohistochemistry, soft agar assay and tumor formation assay in nude mice were applied. Cell cycle distribution was analyzed by flow cytometry. The lower level of PHRF1 mRNA was observed in human lung cancer tissues than that in paracancerous tissues. The decreased expression of PHRF1 protein was observed in H1299 and H1650 cell lines than that in 16HBE and BEAS-2B cell lines. The decreased expression of PHRF1 protein was observed in malignant 16HBE cells compared to control cells. The reduced expression of PHRF1 protein was observed in mice lung tissues treated with BaP than that in control group. Overexpression of PHRF1 inhibited H1299 cell proliferation, colony formation *in vitro* and growth of tumor xenograft *in vivo*, and arrested cell cycle in G1 phase. The decreased expression of TGIF and c-Myc proteins and the increased expression of p21 protein were observed in H1299-PHRF1 cells compared with H1299-pvoid cells. In conclusion, our findings suggest that overexpression of PHRF1 attenuated the proliferation and tumorigenicity of non-small cell lung cancer cell line of H1299.

## INTRODUCTION

Lung cancer was the most common cause of cancer-related death in men and women, and is responsible for 1.8 million deaths in 2012 [1]. Although, various therapeutic methods have been developed, the five-year survival rate is still relatively low [2, 3]. Synergetic effects of genetic alterations, tobacco smoke and environmental factors are considered to drive abnormal gene expression, which contributes to the initiation, development and progression of lung cancer [4–6]. But the molecular mechanisms of lung tumorigenesis have not yet been clearly elucidated.

PHRF1 (PHD and RING finger domain-containing protein 1), also known as RNF221, KIAA1542 or CTD binding SR like protein rA9, which shares significant similarity with the rA9 protein, contains a Ser/Arg-rich domain and binds to the C-terminal domain of the RNA polymerase II large subunit [7]. Previous studies reported two somatic mutations in the PHRF1 gene in breast cancer: one of them is a missense mutation, whereas the other is located within an intron [8, 9]. Recent studies have revealed that PHRF1 is a tumor suppressor in the initiation and development of breast cancer and acute promyelocytic leukemia [10, 11]. PHRF1 could promote the genome integrity by modulating non-homologous end-joining upon DNA damage insults [12]. However, the expression pattern and functional mechanisms of PHRF1 in lung cancer have not yet been elucidated. In this study, we measured the level of PHRF1 expression in human lung cancer tissues, lung cancer cell lines, malignant human bronchial epithelial (16HBE) cells induced by benzo(a)pyrene (BaP) and mice lung tissues treated by BaP. We also investigated the effects of overexpression of PHRF1 on H1299 cell proliferation and tumor formation *in vitro* and *in vivo*, and cell cycle distribution.

## RESULTS

### The level of PHRF1 in human lung cancer tissues

**Figure 1:**
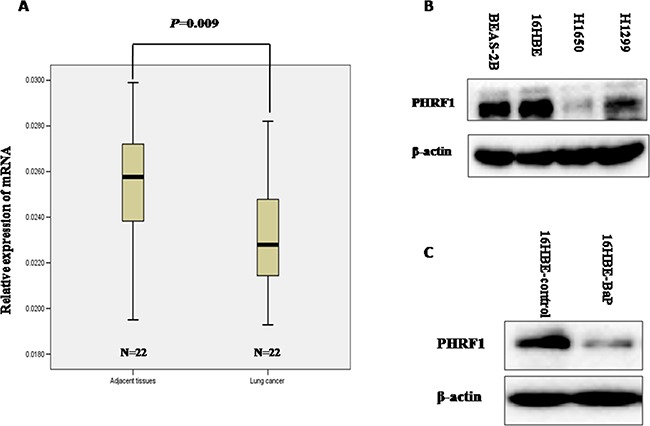
The relative level of PHRF1 mRNA expression in human lung tissues was detected by qRT-PCR and the expression of PHRF1 in lung cancer cell lines and malignant 16HBE cells induced by BaP was detected by western blot The lower level of PHRF1 mRNA expression was observed in human lung cancer tissues than that in paracancerous tissues **A.** The decreased expression of PHRF1 protein was observed in lung cancer cell lines (H1299 and H1650) than that in normal human bronchial epithelial cell lines (16HBE and BEAS-2B) **B.** The decreased expression of PHRF1 protein was observed in malignant 16HBE cells induced by BaP than that in control cells **C.**

To observe the expression of PHRF1 in human lung cancer tissues, we measured the level of PHRF1 mRNA in human lung cancer tissues and its matched paracancerous tissues by quantitative reverse transcriptase polymerase chain reaction (qRT-PCR) assay. Our results showed that the lower level of PHRF1 mRNA expression was observed in human lung cancer tissues than that in paracancerous tissues (*P* = 0.009) (Figure 1A).

### The expression of PHRF1 in lung cancer cell lines and malignant 16HBE cells induced by BaP

The significantly lower level of PHRF1 mRNA expression was observed in human lung cancer tissues than that in paracancerous tissues, thus, we detected the expression of PHRF1 in lung cancer cell lines (H1299 and H1650) and in normal human bronchial epithelial cell lines (16HBE and BEAS-2B) further. Western blot analysis showed that the markedly decreased expression of PHRF1 protein was observed in H1650 and H1299 cell lines compared with 16HBE and BEAS-2B cell lines (Figure 1B). Malignant 16HBE cell line induced by BaP was reported in our previous paper, which could form colonies in soft agar and grow tumor in nude mice [13]. Western blot analysis showed that the dramatically reduced expression of PHRF1 protein was observed in 16HBE-BaP cells as compared to that in 16HBE-control cells (Figure 1C).

### The expression of PHRF1 in mice lung tissues treated by BaP

**Figure 2:**
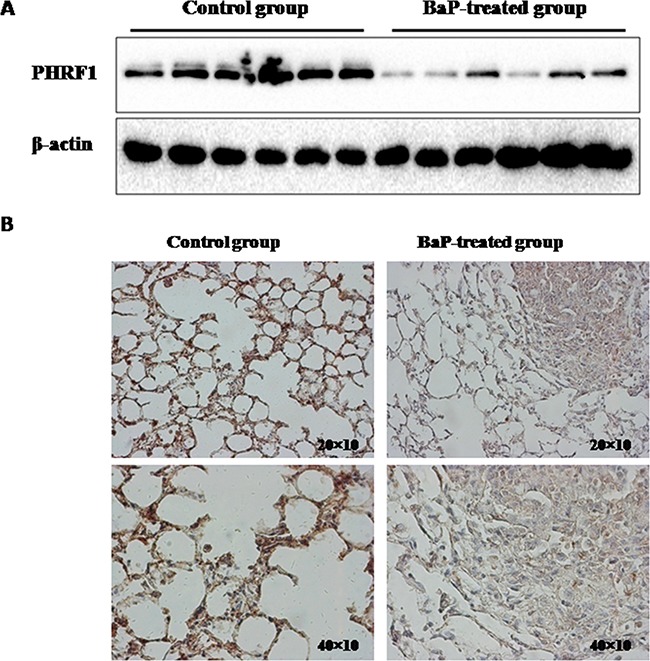
The expression of PHRF1 in BaP-treated mice lung tissues was measured by western blot assay and immunohistochemistry The decreased expression of PHRF1 protein was observed in BaP-treated mice lung tissues compared with control group **A. B.** showed that the staining of PHRF1 was weak in BaP-treated group, but strong in control group.

Above, we observed the markedly reduced expression of PHRF1 in lung cancer from population study and *in vitro* experimental studies as compared to those in its corresponding controls. To further investigate the pattern of PHRF1 expression in *in vivo* experiment, female Kunming mice were treated with BaP to induce lung tumorigenesis. Western blot analysis indicated that the significantly decreased expression of PHRF1 protein was observed in mice lung tissues treated with BaP than that in control group (Figure 2A). The immunohistochemistry assay showed that the staining of PHRF1 was weak in BaP-treated group, but strong in control group (Figure 2B).

### The effects of overexpression of PHRF1 on H1299 cell proliferation

**Figure 3:**
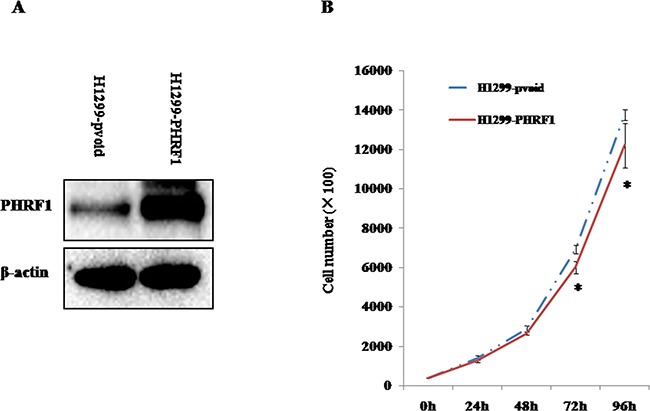
The effects of overexpression of PHRF1 on H1299 cell proliferation **A.** Western blot analysis showed that a stable PHRF1-overexpressed H1299 cell line was established. **B.** Overexpression of PHRF1 inhibited H1299 cell proliferation from 72h. *P<0.05

To observe the effects of overexpression of PHRF1 on the cell proliferation, H1299 cells were infected with PHRF1 lentiviruses. Figure 3A showed that an H1299 cell line stably overexpressing PHRF1 was successfully established. The cell number was counted at different time points. As shown in Figure 3B, the growth of H1299-PHRF1 cells was significantly slower than that of H1299-pvoid cells from 72h (*P* < 0.05).

### The effects of overexpression of PHRF1 on colony formation

**Figure 4:**
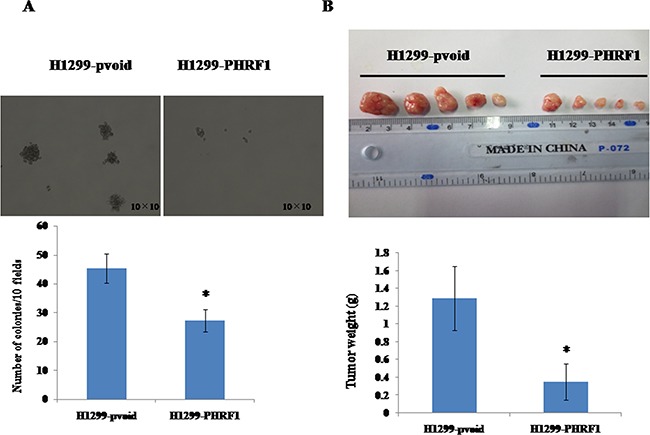
The effects of overexpression of PHRF1 on colony formation and tumor growth in mouse xenograft model The dramatically decreased colony formation was observed in H1299-PHRF1 cells as compared to H1299-pvoid cells **A.** There was a significant reduction in the mean of tumor weight in H1299-PHRF1 cells as compared to H1299-pvoid cells **B.** *P<0.05

Since we observed overexpression of PHRF1 inhibited H1299 cell proliferation, we further observed the effects of overexpression of PHRF1 on colony formation *in vitro*. Figure 4A showed that there was markedly decreased colony formation in H1299-PHRF1 cells (27.2±3.8) as compared to that in H1299-pvoid cells (45.3±5.0) (*P* < 0.05).

### The effects of overexpression of PHRF1 on tumor growth in mouse xenograft model

Above, we observed overexpression of PHRF1 inhibited colony formation of H1299 cells *in vitro*. To assess the role of overexpression of PHRF1 in tumor growth ability *in vivo*, we established BALB/c nude mouse xenograft model with H1299-PHRF1 cells and H1299-pvoid cells by subcutaneous injection. Figure 4B showed that there was a significant decrease in the mean of tumor weight in the group of H1299-PHRF1 cells (0.35±0.21 g) as compared to that in the group of H1299-pvoid cells (1.29±0.36 g) (*P* < 0.05), which suggests that overexpression of PHRF1 significantly inhibited the tumor formation and tumor growth of H1299 cells *in vivo*.

### The effects of overexpression of PHRF1 on the distribution of cell cycle

**Figure 5:**
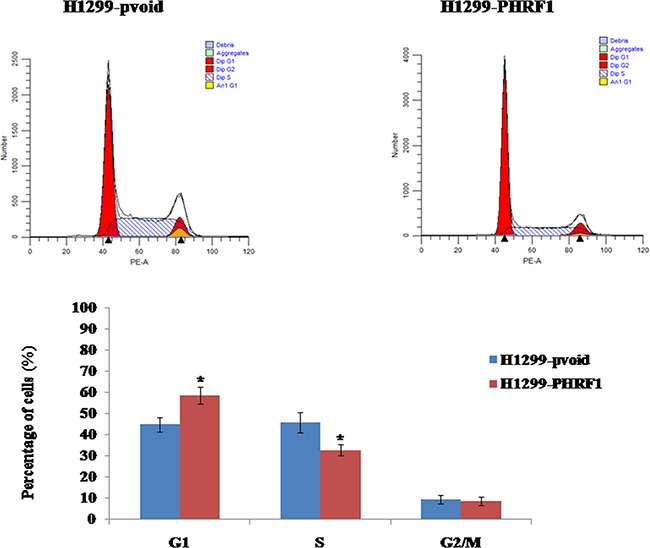
The effects of overexpression of PHRF1 on the distribution of cell cycle Flow cytometry analysis showed that overexpression of PHRF1 increased the cell percentage in G1 phase and decreased the cell percentage in S phase. *P<0.05

We observed overexpression of PHRF1 inhibited H1299 cells proliferation, and cell cycle arrest is one of the major causes of cell growth inhibition. Therefore, flow cytometry assay was conducted to analyze the distribution of cell cycle. Our results showed that the percentage of G1 phase cells was significantly higher in H1299-PHRF1 cells (58.6±3.9%) than that in H1299-pvoid cells (44.8±3.3%) (*P* < 0.05) and the percentage of S phase cells was significant lower in H1299-PHRF1 cells (32.7±2.7%) than that in H1299-pvoid cells (45.8±4.7%) (*P* < 0.05) (Figure 5), which suggests that overexpression of PHRF1 could induce the growth inhibition of H1299 cells by arresting the cell cycle in G1 phase. In addition, we observed that the proliferation index was significantly decreased in H1299-PHRF1 cells (41.4±3.9%) than that in H1299-pvoid cells (55.2±3.3%) (*P* < 0.05).

### Overexpression of PHRF1 upregulated the expression of p21 protein

**Figure 6:**
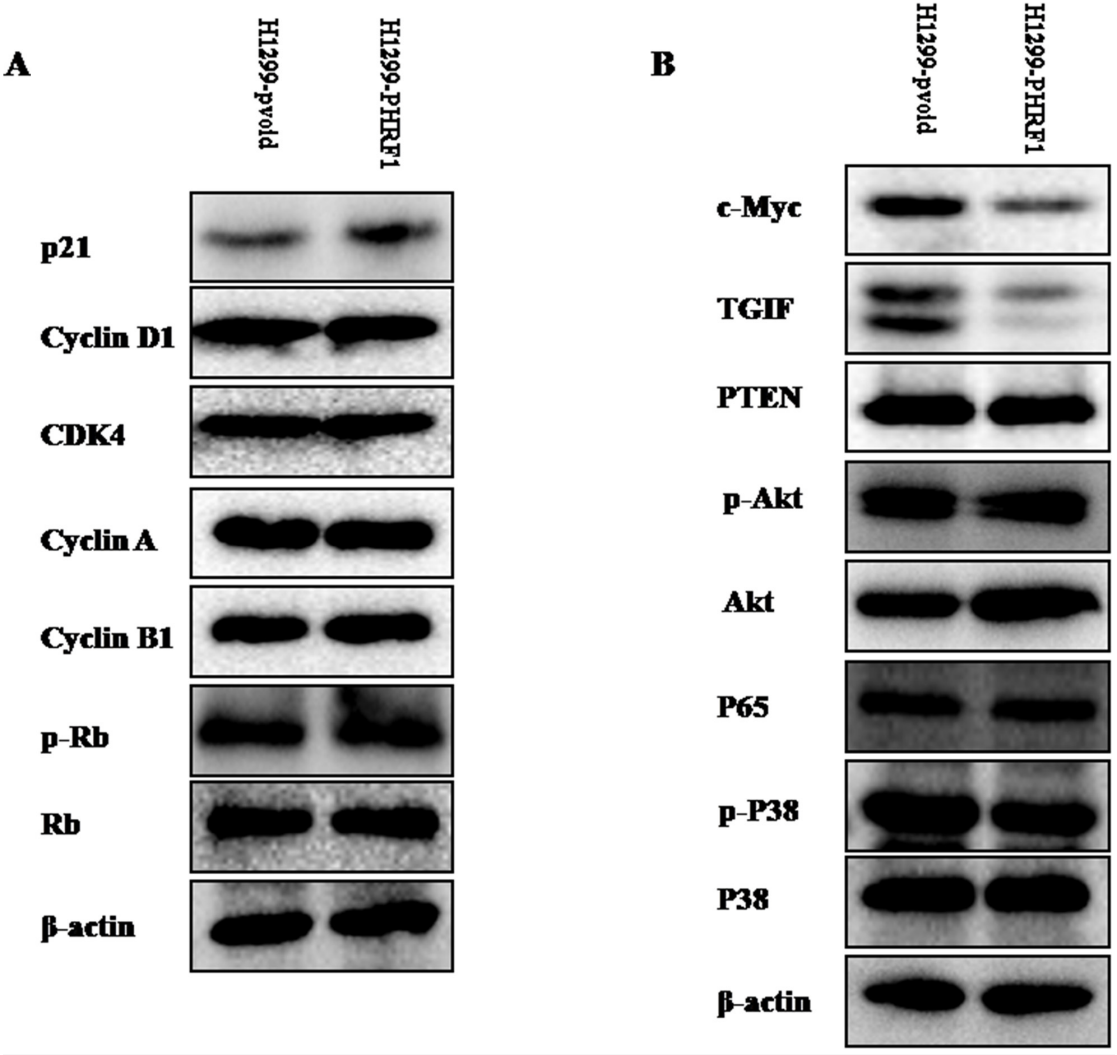
The effects of overexpression of PHRF1 on the expression of interested proteins Western blot analysis showed that overexpression of PHRF1 could increase the expression of p21 protein **A.** and decrease the expression of c-Myc and TGIF proteins **B.**

To explore the mechanism underlying the cell cycle arrest, we detected the expression levels of the cell cycle-related proteins by western blot analysis. As shown in Figure 6A, overexpression of PHRF1 increased the expression of p21 protein in H1299 cells. There were no obvious alterations in the expression of cyclin D1, cyclin A, cyclin B1, CDK4, p-Rb and Rb proteins between H1299-PHRF1 cells and H1299-pvoid cells.

### Overexpression of PHRF1 downregulated the expression of TGIF and c-Myc proteins

We sought to investigate whether or not overexpression of PHRF1 could affect the expression of c-Myc, TGIF, PTEN, p-Akt, Akt, p65, p-p38 and p38 proteins. As shown in Figure 6B, western blot analysis indicated that the markedly decreased expression of TGIF and c-Myc proteins was observed in H1299-PHRF1 cells as compared to those in H1299-pvoid cells. There were no obvious alterations in the expression of p-Akt, Akt, PTEN, p65, p-p38 and p38 proteins between H1299-PHRF1 cells and H1299-pvoid cells.

## DISCUSSION

Previous studies revealed that the PHRF1 gene mapping to 11p15.5 displayed frequent loss of heterozygosity (LOH), somatic mutations or deletion in several human tumors [8–10, 14–17]. A marked decrease in PHRF1 mRNA expression level was detected in human breast cancer samples when compared with adjacent normal tissues [10]. Our results demonstrated the involvement of aberrant expression of PHRF1 in lung tumorigenesis. We observed the decreased expression of PHRF1 mRNA in human lung cancer tissues as compared to that in adjacent non-neoplastic tissues. To confirm the observations in human samples, the expression of PHRF1 protein was measured in lung cancer cells and human bronchial epithelial cells. We also measured the levels of PHRF1 expression in malignant 16HBE cells induced by BaP and mice lung tissues treated with BaP. In consistence with the results from population study, we observed the lower levels of PHRF1 expression in lung cancer cell lines, malignant 16HBE cells induced by BaP and mice lung tissues treated with BaP than those in its corresponding controls, which supports our findings in the cellular levels and animal models to a certain extent.

Aberrant cell proliferation is linked to cancer progression. In this present study, we observed that overexpression of PHRF1 inhibited H1299 cell proliferation. To further observe the effects of overexpression of PHRF1 on tumor formation *in vitro* and *in vivo*, soft agar assay and mouse xenograft model were conducted. Our results showed that overexpression of PHRF1 decreased the ability of tumor formation and growth of H1299 cells *in vitro* and *in vivo*. This observation confirmed the notion that the aberrant expression of PHRF1 might be involved in lung tumorigenicity. Previous study showed that restoration of PHRF1 expression suppressed the capability of tumor formation and growth of breast cancer cell line in *in vitro* and *in vivo* experiments [10]. Prunier et al. reported that restoration of PHRF1 activity could suppress the tumor formation of acute promyelocytic leukemia in *in vivo* experiments [11]. Taken together, these findings suggest that PHRF1 may play a tumor-suppression function in lung tumorigenicity.

To our knowledge, cell cycle arrest is one of the major causes of cell growth inhibition. To observe the effects of overexpression of PHRF1 on the distribution of cell cycle of H1299 cells, flow cytometry analysis was performed. Our results showed that the cell population of G1 phase was dramatically higher in H1299-PHRF1 cells than that in H1299-pvoid cells, which suggests that overexpression of PHRF1 induced G1 phase cell cycle arrest. Accompanied with G1 phase cell cycle arrest, we further observed the increased expression of p21 protein in H1299-PHRF1 cells compared with H1299-pvoid cells. Studies have shown that p21 is one of the major cyclin-dependent kinase inhibitors (CDKIs). It negatively regulates the cell cycle progression and arrests proliferation by binding to active cyclin-CDK (cyclin-dependent kinase) complex and inhibiting their kinase activities [18–21], thereby decreasing the level of hyper-phasphorylated Rb (p-Rb) [22]. But in this present study, we did not observe obvious change of p-Rb between H1299-PHRF1 cells and H1299-pvoid cells.

TGIF (TG-interacting factor) is a transcriptional repressor within the transforming growth factor β (TGF-β) and retinoic acid (RA) signaling pathway. It functions as an oncogenic protein involving in the initiation, development and progression of tumors including lung cancer [13, 23]. Castro et al. reported that aberrant methylation of TGIF had been detected in lung tumors [24]. Wang et al. reported that elevated expression of TGIF was involved in lung carcinogenesis [13]. Xiang et al. reported that TGIF promoted the growth and migration of cancer cells in non-small cell lung cancer [23]. In this present study, we observed that overexpression of PHRF1 markedly decreased the expression of TGIF protein, which suggests PHRF1 may regulate the expression of TGIF in non-small cell lung cancer cell line. One published paper reported that PHRF1 interacted with TGIF and enforced TGIF decay by driving its ubiquitination at lysine 130 [10]. Both of these two studies confirmed the regulation of TGIF expression by PHRF1.

The c-Myc oncoprotein is a pleiotropic transcription factor that has been shown to regulate a great number of genes and to be involved in many physiological functions, such as proliferation, cell cycle, apoptosis, differentiation, angiogenesis, metabolism and microRNA regulation [25, 26]. The c-Myc oncogene is frequently overexpressed in lung cancer [27]. In this study, we found that overexpression of PHRF1 reduced the expression of c-Myc protein in H1299 cells. However, the mechanism of PHRF1 regulating the expression of c-Myc protein in non-small cell lung cancer cell was not addressed in this study, which should be focused on in the future study.

Although the findings are very encouraging, there are several limitations in this current study. Firstly, PHRF1-overexpressed cell model was applied to address the potential role of PHRF1 in lung tumorigenesis in this study. PHRF1-silenced cell model should be performed to verify our findings in the future study. Secondly, only 22 human lung cancer tissue samples and its matched paracancerous tissue samples were used to measure the expression of PHRF1 in the mRNA level in this present study. Studies with larger sample sizes should be carried out to verify our findings. Thirdly, there are several types of lung cancers that depend on different genetic and epigenetic alterations. Thus, future studies should pay more attention to the potential role of PHRF1 in specific type of lung cancers. In addition, only one cell line was applied to address the effects of PHRF1 overexpression on the proliferation and tumorigenicity of non-small cell lung cancer cell. Other stable PHRF1-overexpressed non-small cell lung cancer cell lines should be constructed to verify the findings in the future studies.

In conclusions, our findings suggest that aberrant PHRF1 expression may be involved in lung tumorigenesis. Overexpression of PHRF1 attenuated the tumorigenicity of H1299 cells and induced the growth inhibition of H1299 cells through G1 phase cell cycle arrest by upregulating p21. Therefore, this study extends our knowledge of the lung tumorigenesis.

## MATERIALS AND METHODS

### Cell culture

16HBE cell was gifted from Prof. Wen Chen (Sun Yat-sen University). Malignant 16HBE cell line induced by BaP (16HBE-BaP) and its control cell (16HBE-control) were kept in our laboratory [13]. 16HBE cell, 16HBE-BaP cell and 16HBE-control cell were maintained in minimum essential medium (MEM) supplemented with penicillin (100 U/ml), streptomycin (100 μg/ml), 10% fetal bovine serum (FBS) and 2 mM L-glutamine. HEK293T, H1299 and H1650 cell lines were obtained from the Cell Resource Center, Peking Union Medical College (which is the headquarters of National Infrastructure of Cell Line Resource, NSTI). BEAS-2B cell was obtained from Shanghai Bogoo Biology. BEAS-2B, H1299 and H1650 were cultured in RPMI-1640 supplemented with penicillin (100 U/ml), streptomycin (100 μg/ml), 10% FBS and 2 mM L-glutamine. HEK293T was cultured in DMEM supplemented with penicillin (100 U/ml), streptomycin (100 μg/ml), 10% FBS and 2 mM L-glutamine. All of the cell lines were incubated at 37°C in a humidified atmosphere containing 5% CO_2_.

### Lentivirus production

The packaging vector pCMVdeltaR8.91, the VSV-G envelope glycoprotein vector pMD.G, and the full-length of PHRF1 in the pLenti6/V5 vector were gifted from Prof. Chang (Institute of Biochemical Sciences, National Taiwan University) and were co-transfected into HEK293T cells. After 48h incubation, the viral particles were collected to infect H1299 cells in the presence of polybrene (8 μg/ml) for viral infection [12]. Stable clones were selected with 8 μg/ml of blasticidin (Sigma, USA) for 21 days and named as H1299-PHRF1. Cells infected with empty vector viral particles were named as H1299-pvoid.

### Biological samples

Human lung cancer tissue samples and its matched paracancerous tissue samples were mentioned in our previous study [13]. Written informed consent for participation in this study was obtained from all patients prior to the surgical operations. This study was approved by the Ethics Committee of the First Affiliated Hospital of Zhengzhou University and the associated methods were carried out in accordance with the approved guidelines. BaP-treated mice lung tissues and control mice lung tissues were maintained in our laboratory [13].

### Quantitative reverse transcriptase polymerase chain reaction

Total RNA was isolated using RNAiso Plus (Takara, Japan). Reverse transcriptase PCR was performed using the reverse transcriptase kit from Takara (Prime ScriptH RT reagent Kit-Perfect Real Time). Quantitative real time polymerase chain reaction was done using the SYBR Green PCR Master Mix from Takara (Premix Ex Taq^TM^-Perfect Real Time) in a total volume of 20 μl on the 7300 Real-Time PCR System (Applied Biosystems): 95°C for 30s, 40 cycles of 95°C for 5s, 60°C for 30s. Experiments were repeated in triplicates. The relative level of gene expression was represented as 2^−^^ΔCt^ (ΔCt=Ct_PHRF1_-Ct_GAPDH_). The primers were used to amplify the human PHRF1 gene as follows: PHRF1-forward, 5′- CCAATCTGTCTCAACGCATTC -3′; PHRF1-reverse, 5′- GACAGGAATTGGCATTCTTGG -3′. GAPDH was used as the reference gene (GAPDH-forward, 5′- GACCCCTTCATTGACCTCAAC-3′; GAPDH-reverse, 5′-CTTCTCCATGGTGGTGAAGA-3′).

### Measurement of H1299-PHRF1 and H1299-pvoid cells proliferation

H1299-PHRF1 cells and H1299-pvoid cells were seeded in triplicate at a density of 4 × 10^4^ cells per well in 12-well plates. Cells were harvested and counted at 24h, 48h, 72h and 96h after seeding by a CASY Cell Counter (Scharfe System, Reutlingen, Germany).

### Soft agar assay

Soft agar assay was applied to evaluate the abilities of H1299-PHRF1 cells and H1299-pvoid cells to grow as anchorage-independent colonies. 2 ml of 0.6% low melting point agarose in RPMI-1640 medium containing 10% FBS were poured into 6-well plate and allowed to solidify at room temperature. After solidification, 500 cells were suspended in 1 ml of 0.35% low melting point agarose in the same medium and then plated on top of the base layer (3 wells per group). The cells were cultured for 14 days. Colonies with at least 50 cells were counted using a microscope at 100× magnification and the number of colonies in soft agar (10 fields per well) was quantified [13].

### Tumor formation assay in nude mice

All experimental procedures involving animals were performed in accordance with the guideline for the Care and Use of Laboratory Animals. This work was approved by the Ethics Committee of Henan Center for Disease Control and Prevention. Four-week old female BALB/c nude mice were purchased from Vital River Laboratory Animal Technology Co. Ltd (Beijing, China). H1299-PHRF1 cells and H1299-pvoid cells were harvested, washed twice with PBS, and suspended in PBS. Five mice per group received 5 × 10^6^ cells in 200 μl of PBS injected subcutaneously into the back neck of each mouse. The mice were housed in a specific pathogen-free environment and sacrificed at 21 days post-injection [13].

### Cell cycle assay

H1299-PHRF1 cells and H1299-pvoid cells were seeded in 60-mm plate. Cells were detached by trypsinization when growing to 70%-80% confluence, washed twice with cold PBS, fixed in 70% ethanol at −20°C overnight. The fixed cells were washed twice with cold PBS, suspended in 0.5 ml of PBS containing 100 μg/ml of RNase (Invitrogen Life Technologies, USA) and 50 μg/ml of propidium iodide (PI) (Sigma, USA) and incubated at room temperature for 40 min in the dark. Cell cycle analysis was performed by flow cytometry (BD Biosciences). Duplicates were performed in all experiments and experiments were performed on two occasions.

### Western blot assay

The total proteins were extracted with lysis buffer solution supplemented with protease inhibitors and phosphatase inhibitors (Pierce, USA) and quantified by the BCA protein assay (Pierce, USA). Each sample (30 μg) was separated by 10% SDS-PAGE, and then transferred to nitrocellulose (NC) membranes (PALL, USA). After blocking with 5% BSA in Tris-buffered saline-Tween 20, the membrane was incubated with primary antibody for overnight at 4°C. PHRF1 (ab85974) was purchased from Abcam. Akt (sc-8312), p-Akt (sc-33437), TGIF (sc-9084), p38 (sc-535), p-p38 (sc-17852-R), p21 (sc-397), cyclin A (sc-751), cyclin B1 (sc-752), cyclin D1 (sc-718) and CDK4 (sc-260) were purchased from Santa Cruz Technology. Rb (#9313S), p-Rb (#8516S), c-Myc (#13987S), p65 (#8242S) and PTEN (#9188S) were purchased from Cell Signaling Technology. After incubation with peroxidase-coupled anti-rabbit-IgG (ZSGB-BIO, Beijing, China) at room temperature for 1h, the protein bands were visualized by Bio-Rad Clarity™ western ECL substrate (Bio-Rad, USA) in the ChemiDoc™ XRS+ Imaging System (Bio-Rad, USA). Antibody to β-actin (1:1000, Santa Cruz Technology, sc-8432) was used as a loading control.

### Immunohistochemistry

Immunohistochemistry analysis was performed according to standard procedures (Beijing ComWin Biotech Co., Ltd; CW0120).

### Statistical analysis

Data were displayed as mean ± standard deviation (SD) or median. SPSS 13.0 software (SPSS, Chicago, IL) was used to estimate the statistical significance between groups. Data were analyzed by Student's *t* test and Mann-Whitney test. All the tests were two-sided, the *P* value of less than 0.05 was considered to be statistical significance.

## References

[1] Torre LA, Bray F, Siegel RL, Ferlay J, Lortet-Tieulent J, Jemal A (2015). Global cancer statistics 2012. CA Cancer J Clin.

[2] Parkin DM, Bray F, Ferlay J, Pisani P (2001). Estimating the world cancer burden: Globocan 2000. Int J Cancer.

[3] DeSantis CE, Lin CC, Mariotto AB, Siegel RL, Stein KD, Kramer JL, Alteri R, Robbins AS, Jemal A (2014). Cancer treatment and survivorship statistics 2014. CA Cancer J Clin.

[4] Cooper WA, Lam DC, O'Toole SA, Minna JD (2013). Molecular biology of lung cancer. J Thorac Dis.

[5] Schwartz A, Cote M (2016). Epidemiology of Lung Cancer. Adv Exp Med Biol.

[6] Liu Z, Mai C, Yang H, Zhen Y, Yu X, Hua S, Wu Q, Jiang Q, Zhang Y, Song X, Fang W (2014). Candidate tumour suppressor CCDC19 regulates miR-184 direct targeting of C-Myc thereby suppressing cell growth in non-small cell lung cancers. J Cell Mol Med.

[7] Salloum R, Franek BS, Kariuki SN, Rhee L, Mikolaitis RA, Jolly M, Utset TO, Niewold TB (2010). Genetic variation at the IRF7/PHRF1 locus is associated with autoantibody profile and serum interferon-alpha activity in lupus patients. Arthritis Rheum.

[8] Ellis MJ, Ding L, Shen D, Luo J, Suman VJ, Wallis JW, Van Tine BA, Hoog J, Goiffon RJ, Goldstein TC, Ng S, Lin L, Crowder R (2012). Whole-genome analysis informs breast cancer response to aromatase inhibition. Nature.

[9] Nik-Zainal S, Alexandrov LB, Wedge DC, Van Loo P, Greenman CD, Raine K, Jones D, Hinton J, Marshall J, Stebbings LA, Menzies A, Martin S, Leung K (2012). Mutational processes molding the genomes of 21 breast cancers. Cell.

[10] Ettahar A, Ferrigno O, Zhang MZ, Ohnishi M, Ferrand N, Prunier C, Levy L, Bourgeade MF, Bieche I, Romero DG, Colland F, Atfi A (2013). Identification of PHRF1 as a tumor suppressor that promotes the TGF-beta cytostatic program through selective release of TGIF-driven PML inactivation. Cell Rep.

[11] Prunier C, Zhang MZ, Kumar S, Levy L, Ferrigno O, Tzivion G, Atfi A (2015). Disruption of the PHRF1 Tumor Suppressor Network by PML-RARalpha Drives Acute Promyelocytic Leukemia Pathogenesis. Cell Rep.

[12] Chang CF, Chu PC, Wu PY, Yu MY, Lee JY, Tsai MD, Chang MS (2015). PHRF1 promotes genome integrity by modulating non-homologous end-joining. Cell Death Dis.

[13] Wang Y, Wang H, Gao H, Xu B, Zhai W, Li J, Zhang C (2015). Elevated expression of TGIF is involved in lung carcinogenesis. Tumour Biol.

[14] Winqvist R, Hampton GM, Mannermaa A, Blanco G, Alavaikko M, Kiviniemi H, Taskinen PJ, Evans GA, Wright FA, Newsham I, Cavenee WK (1995). Loss of heterozygosity for chromosome 11 in primary human breast tumors is associated with poor survival after metastasis. Cancer Res.

[15] Ali IU, Lidereau R, Theillet C, Callahan R (1987). Reduction to homozygosity of genes on chromosome 11 in human breast neoplasia. Science.

[16] Jonas RE, Kimonis VE (2001). Chest wall hamartoma with Wiedemann-Beckwith syndrome: clinical report and brief review of chromosome 11p15 5-related tumors. Am J Med Genet.

[17] Karnik P, Paris M, Williams BR, Casey G, Crowe J, Chen P (1998). Two distinct tumor suppressor loci within chromosome 11p15 implicated in breast cancer progression and metastasis. Hum Mol Genet.

[18] Grana X, Reddy EP (1995). Cell cycle control in mammalian cells: role of cyclins cyclin dependent kinases (CDKs) growth suppressor genes and cyclin-dependent kinase inhibitors (CKIs). Oncogene.

[19] Toyoshima H, Hunter T (1994). p27 a novel inhibitor of G1 cyclin-Cdk protein kinase activity is related to p21. Cell.

[20] Kagawa S, Fujiwara T, Hizuta A, Yasuda T, Zhang WW, Roth JA, Tanaka N (1997). p53 expression overcomes p21WAF1/CIP1-mediated G1 arrest and induces apoptosis in human cancer cells. Oncogene.

[21] Munoz-Alonso MJ, Acosta JC, Richard C, Delgado MD, Sedivy J, Leon J (2005). p21Cip1 and p27Kip1 induce distinct cell cycle effects and differentiation programs in myeloid leukemia cells. J Biol Chem.

[22] Denicourt C, Dowdy SF (2004). Cip/Kip proteins: more than just CDKs inhibitors. Genes Dev.

[23] Xiang G, Yi Y, Weiwei H, Weiming W (2015). TGIF1 promoted the growth and migration of cancer cells in nonsmall cell lung cancer. Tumour Biol.

[24] Castro M, Grau L, Puerta P, Gimenez L, Venditti J, Quadrelli S, Sanchez-Carbayo M (2010). Multiplexed methylation profiles of tumor suppressor genes and clinical outcome in lung cancer. J Transl Med.

[25] Chen BJ, Wu YL, Tanaka Y, Zhang W (2014). Small molecules targeting c-Myc oncogene: promising anti-cancer therapeutics. Int J Biol Sci.

[26] Sun XX, He X, Yin L, Komada M, Sears RC, Dai MS (2015). The nucleolar ubiquitin-specific protease USP36 deubiquitinates and stabilizes c-Myc. Proc Natl Acad Sci U S A.

[27] Miao LJ, Huang SF, Sun ZT, Gao ZY, Zhang RX, Liu Y, Wang J (2013). MiR-449c targets c-Myc and inhibits NSCLC cell progression. FEBS Lett.

